# A joint analysis to identify loci underlying variation in nematode resistance in three European sheep populations

**DOI:** 10.1111/jbg.12071

**Published:** 2014-01-08

**Authors:** V Riggio, R Pong-Wong, G Sallé, MG Usai, S Casu, CR Moreno, O Matika, SC Bishop

**Affiliations:** 1 The Roslin Institute and R(D)SVS, University of EdinburghEaster Bush, Midlothian, UK; 2 Station d'Amélioration Génétique des Animaux, INRA, UR631Castanet-Tolosan, France; 3 Settore Genetica e Biotecnologie, AGRIS SardegnaOlmedo, Sassari, Italy

**Keywords:** Joint analysis, nematode resistance, regional heritability mapping, sheep, SNP

## Abstract

Gastrointestinal nematode infections are one of the main health/economic issues in sheep industries, worldwide. Indicator traits for resistance such as faecal egg count (FEC) are commonly used in genomic studies; however, published results are inconsistent among breeds. Meta (or joint)-analysis is a tool for aggregating information from multiple independent studies. The aim of this study was to identify loci underlying variation in FEC, as an indicator of nematode resistance, in a joint analysis using data from three populations (Scottish Blackface, Sarda × Lacaune and Martinik Black-Belly × Romane), genotyped with the ovine 50k SNP chip. The trait analysed was the average animal effect for *Strongyles* and *Nematodirus* FEC data. Analyses were performed with regional heritability mapping (RHM), fitting polygenic effects with either the whole genomic relationship matrix or matrices excluding the chromosome being interrogated. Across-population genomic covariances were set to zero. After quality control, 4123 animals and 38 991 SNPs were available for the analysis. RHM identified genome-wide significant regions on OAR4, 12, 14, 19 and 20, with the latter being the most significant. The OAR20 region is close to the major histocompatibility complex, which has often been proposed as a functional candidate for nematode resistance. This region was significant only in the Sarda × Lacaune population. Several other regions, on OAR1, 3, 4, 5, 7, 12, 19, 20 and 24, were significant at the suggestive level.

## Introduction

Gastrointestinal nematode infections are one of the main health issues in grazing ruminants and have a great impact on sheep industries, worldwide. To investigate the observed variation in nematode resistance indicator traits such as faecal egg count (FEC), and to potentially provide genetic markers that may be used for selection, several quantitative trait loci (QTL) studies have addressed nematode resistance. However, so far, little overall consensus has emerged from these studies in terms of resistance loci, using either microsatellite-based linkage analyses (LA) (e.g. Crawford *et al.* 2006; Davies *et al.* 2006) or genome-wide association (GWA) analyses (e.g. Kemper *et al.* 2011; Sallé *et al.* 2012; Riggio *et al.* 2013). This may be due to both the apparent genetic complexity of the trait and the fact that these studies are very diverse, involving a variety of sheep breeds, nematode species and experimental approaches.

We propose that there are statistical techniques which can be used to jointly analyse these diverse datasets and hence gain greater power. These are based on the concept of meta-analysis, a tool for aggregating information from multiple independent studies (Cochran 1954; Fleiss 1993). The use of meta-analysis is becoming popular in GWA studies, because one can virtually collect data from tens of thousands of individuals, and this provides sufficient power to identify variants even with small effect sizes (de Bakker *et al.* 2008; Zeggini & Ioannidis 2009). In humans, several large-scale meta-analyses have been performed for diseases including type 1 diabetes (Barrett *et al.* 2009), bipolar disorder (Scott *et al.* 2009) and Crohn's disease (Franke *et al.* 2010), and these analyses have identified associations not revealed in the individual studies. Meta-analysis studies have been also conducted in livestock, such as in cattle (Khatkar *et al.* 2004) and pigs (Switonski *et al.* 2010). Generally, these meta-analyses utilize public domain information on the trait/species considered and appropriately combine the output results to get overall p-values for each marker or QTL.

We are in the fortunate position of having direct access to datasets comprising three different populations [Scottish Blackface (SBF), Sarda × Lacaune backcross (SAR) and Martinik Black-Belly × Romane backcross (MBR)], each of which has nematode resistance phenotypes as well as genotypes from the ovine 50k SNP chip. Therefore, instead of meta-analysis techniques combining published results, we were able to carry out a joint analysis considering the three populations together. Therefore, the aim of this study was to identify genomic regions underlying variation in FEC in a joint analysis which assumed the three populations to be genetically unrelated.

## Materials and methods

### Populations

Three populations were used for the analysis. As shown in the principal component plot of SNP chip markers reported in Figure S1, the three populations are genetically very distant and hence can be considered to be unrelated. The SBF population comprised 752 F2 and double backcross lambs from two lines selected for carcass lean content from the same foundation population, bred from 10 sires (half-sib family size = 11–146). More details on the data structure are given in Riggio *et al.* (2013). The SAR population consisted of 2371 ewes, derived from 927 backcross Sarda × Lacaune ewes subsequently crossed to purebred Sarda rams. A detailed description of the animals is given in Sechi *et al.* (2009). The MBR population consisted of 1000 backcross lambs, obtained from mating Martinik Black-Belly × Romane F1 rams with purebred Romane ewes. The experimental design is described in Sallé *et al.* (2012). In total, 4123 animals were included in the final dataset.

### Phenotypic measurements

Faecal samples were collected from the rectum of the animals at different ages for the different populations, assuming that FEC at different ages is genetically correlated (Bishop *et al.* 1996; Goldberg *et al.* 2012), and FEC was determined on each sample. The SBF population comprised growing lambs facing natural (mixed species) challenge at pasture; the MBR comprised growing lambs facing deliberate *Haemonchus contortus* challenge; and the SAR population comprised lactating ewes facing natural (mixed species) challenge at pasture. Eggs collected in the SBF population were classified according to whether they were *Nematodirus* spp. or other nematode genera collectively termed *Strongyles*, the latter potentially including *Oesophagostomum*, *Chabertia*, *Bunostomum*, *Trichostrongylus*, *Cooperia*, *Ostertagia*, *Teladorsagia* and *Haemonchus* genera. The SAR and MBR populations' eggs were classified only as *Strongyles* FEC.

Prior to analysis, FEC measurements were log-transformed to get an approximation to the normal distribution. Moreover, to avoid the problems related to the (environmental) heterogeneity of the data (e.g. different time points, different fixed effects), instead of using time-point-specific data, averaged FEC data across the time points for each population were analysed (both *Nematodirus* and *Strongyles*). This ‘average animal effect’ for each trait was estimated by fitting a repeatability model to trait values across the different time points and then standardized to a mean of 0 and a standard deviation of 1. The fixed effects considered in the analyses were as follows: sex, year of birth, management group, litter size and age of dam, with day of birth as covariate for SBF (Riggio *et al.* 2013); sex, management group, litter size and age at infection for MBR (Sallé *et al.* 2012); and sampling date, litter size, age at lambing, group and physiological state (dry-off, periparturient, lactation) for SAR.

### Genotype data

Animals were genotyped using the 50k SNP chip. The SNP genotype data were subjected to quality control (QC) measures, specific for each population (see Data S1). Furthermore, markers on the sex chromosome were removed from the analysis. After QC, 42 841 SNPs were available for the SBF population, 44 859 for the SAR and 42 469 for the MBR. Out of these SNPs, 38 991 were in common among the three populations and therefore used for further analyses. Positions of SNP markers were obtained from the Sheep Genome browser v2.0 (http://www.livestockgenomics.csiro.au/sheep/).

### Statistical analyses

Analyses were performed using the regional heritability mapping (RHM) approach (Nagamine *et al.* 2012), in which each chromosome (OAR) is divided into windows of a predefined number of SNPs, and the variance attributable to each window estimated. In our case, the window size was 100 adjacent SNPs, and the window was shifted every 50 SNPs. Initially, the analyses were performed using only the *Strongyles* data. Subsequently, a second analysis was performed substituting the *Nematodirus* FEC data for the *Strongyles* data in the SBF population, to test whether common QTL regions across populations could be detected when combining data from very different nematode species.

A mixed model was used for the analysis, with residual and additive genetic (both regional genomic and whole genomic) effects fitted as random. The whole genomic additive effect was estimated using the genomic relationship (**G**) matrix constructed from all SNPs, but modified as described below, whereas the regional genomic additive effect was estimated from a genomic relationship matrix constructed from the SNPs within each window, that is, region. Whole genomic, regional genomic and residual variances were 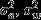 and 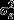, respectively. Phenotypic variance, 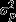, was then given by 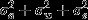. Whole genomic heritability was estimated as 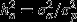, whereas the regional heritability was 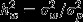.

Genomic relationship matrices comprise identity-by-state (IBS) relationships between all animals. However, because the three populations are distant and hence linkage phases between marker and causative mutation are likely to differ between populations, it is difficult to assume that across-population IBS relationships contain useful information on genetic relatedness or QTL effects. Further, including such covariances risks including spurious noise effects. Therefore, to take into account the observation that the populations are genetically distant, the **G** matrix was set to be block diagonal by population.

Moreover, to account for the population structure, that is, each population comprising rather few sire families and hence long chromosome segments inherited intact leading to long stretches of LD, we ran further analyses with **G** matrices created separately for each chromosome under investigation, always excluding the chromosome being interrogated (i.e. 26 different **G** matrices were considered). We termed this the ‘n-1’ **G** matrix. This avoided QTL effects on individual chromosomes being absorbed by the overall **G** matrix.

To test for the differences in regional variance, a likelihood ratio test (LRT) was used to compare a model fitting variance in a specific window (fitting both whole-genome and region-specific additive variance) against the null hypothesis of no variance in that window (whole-genome additive variance only). The test statistic was assumed to follow a mixture of 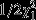 and 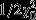 distributions (Self & Liang 1987). In total, 788 windows were tested, of which half were used in the Bonferroni correction, to account for the overlapping windows. Hence, after Bonferroni correction to account for multiple testing, the LRT thresholds were 13.38 and 9.11 corresponding to p-values of p < 1.27 × 10^−4^ and p < 2.54 × 10^−3^ (i.e. –log_10_(p) of 3.90 and 2.60), for genome-wide (p < 0.05) and suggestive significance (i.e. one false positive per genome scan) levels, respectively.

## Results

The heritability estimated for the *Strongyles* FEC phenotype was 0.35 when using the whole **G** matrix. Further, when using any of the n-1 **G** matrices, essentially the same value was invariably obtained. However, the heritability estimate was higher (0.39) for the analysis in which *Nematodirus* FEC for SBF population and *Strongyles* FEC for SAR and MBR populations were combined.

**Figure 1:**
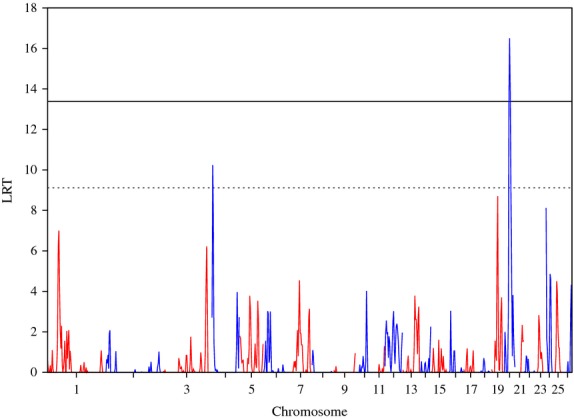
Plot of the likelihood ratio test (LRT) across the genome, considering the whole genomic relationship matrix for *Strongyles* average animal effect. Genome-wide p < 0.05 (solid line) and suggestive (dashed line) thresholds are also shown.

Likelihood ratio test results obtained when using the whole **G** matrix for *Strongyles* average animal effect are presented in Figure 1. Three regions on OAR 20 reached the genome-wide significance level, and one on OAR 4 the suggestive level. However, the heritabilities attributable to these regions were quite low, being 0.02 for the genome-wide significant regions and 0.01 for the suggestive one (Table 1). Notably, some QTL which had previously been found to be highly significant, such as OAR12 in the MBR population, were not significant in this analysis.

**Table 1:** Regional heritability (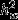) considering the whole genomic relationship matrix for *Strongyles* average animal effect, for windows significant both at the genomic level (p < 0.05) and at the suggestive level

OAR	Window	SNP and position (in bp)		LRT	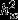
		Start	End		
20	8	s75212.1 21544707	OAR20_29673753.1 27071832	13.78	0.02
20	9	OAR20_25310480.1 24023600	s02808.1 30879395	16.50	0.02
20	10	OAR20_29777242_X.1 27178392	OAR20_37979703.1 34257323	13.88	0.02
4	2	OAR4_3821431.1 3731353	OAR4_11023787.1 10238576	10.24	0.01

**Figure 2:**
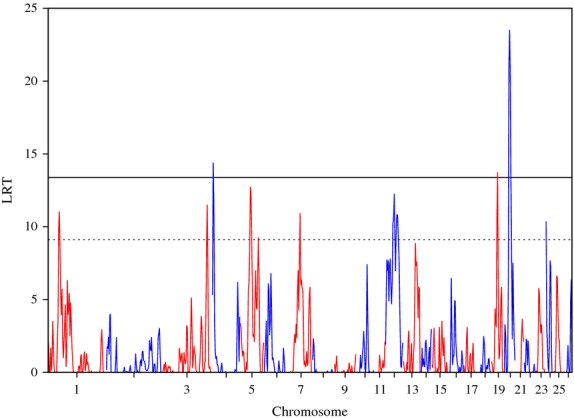
Plot of the likelihood ratio test (LRT) across the genome, considering the n-1 chromosome genomic relationship matrix for *Strongyles* average animal effect. Genome-wide p < 0.05 (solid line) and suggestive (dashed line) thresholds are also shown.

When using the n-1 **G** matrix, five regions significant at the genome-wide level and 21 regions significant at the suggestive level were identified across the genome (Figure 2). Of these, the 4 regions already significant in the previous analysis were confirmed as such, with the region on OAR 4 reaching the genome-wide significance level (LRT = 14.40). In addition, a new region on OAR 19 was also identified as significant at the genome-wide level (LRT = 13.74). However, the heritability for these regions was always low (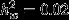), that is, these regions explain only a small part of the genetic variation in the trait analysed. Several regions that reached the suggestive significance level (on OAR 1, 3, 4, 5, 7, 12, 19, 20 and 24) had low heritability estimates ranging between 0.01 and 0.02 (Table 2). It should be noted that the n-1 **G** matrix approach allowed identification of some of the regions of interest that were previously found in individual populations, including the OAR12 QTL described above.

**Table 2:** Regional heritability (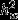) considering the n-1 chromosome genomic relationship matrix for *Strongyles* average animal effect, for windows significant both at the genomic level (p < 0.05) and at the suggestive level

OAR	Window	SNP and position (in bp)		LRT	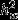
		Start	End		
4	2	OAR4_3821431.1 3731353	OAR4_11023787.1 10238576	14.40	0.02
19	10	OAR19_31723606.1 30252049	OAR19_38703754.1 37090315	13.74	0.02
20	8	s75212.1 21544707	OAR20_29673753.1 27071832	21.28	0.02
20	9	OAR20_25310480.1 24023600	s02808.1 30879395	23.52	0.02
20	10	OAR20_29777242_X.1 27178392	OAR20_37979703.1 34257323	20.74	0.02
1	17	OAR1_51923225.1 49582895	OAR1_59033021.1 55894733	9.88	0.01
1	18	OAR1_55194024.1 52549191	s57947.1 59090067	11.04	0.01
3	69	OAR3_217545698.1 201928340	s47288.1 208579395	11.50	0.01
4	3	OAR4_7316477.1 6895493	OAR4_13768293.1 12959670	11.70	0.01
5	16	OAR5_52221425.1 48431395	OAR5_58968886.1 54639928	12.74	0.01
5	17	OAR5_55493305.1 51654379	s06427.1 59032331	12.34	0.01
5	18	OAR5_59030457.1 54712950	OAR5_67400660.1 61737280	9.48	0.01
5	28	OAR5_92603004.1 84918234	OAR5_98355816.1 90702581	9.26	0.01
7	16	OAR7_47757823.1 42931982	OAR7_53605138.1 48351746	10.94	0.01
12	12	OAR12_37241680.1 32919247	OAR12_43103127.1 38396797	10.16	0.01
12	13	OAR12_39896412.1 35380248	OAR12_46545889.1 41455928	10.66	0.01
12	14	OAR12_43172807.1 38463864	s74913.1 44383517	12.26	0.01
12	15	s04690.1 41497406	s21761.1 47009181	9.48	0.01
12	17	s71572.1 47106106	OAR12_59862156.1 53837493	9.80	0.01
12	18	s21970.1 50082089	OAR12_63101278.1 56700502	10.82	0.01
12	19	OAR12_60135274.1 54027615	s50610.1 59498519	10.80	0.01
12	20	OAR12_63132677.1 56732372	OAR12_68810185.1 62292818	9.32	0.01
19	9	OAR19_28888123.1 27364542	OAR19_35714124.1 34193643	10.64	0.01
20	11	OAR20_34312897.1 30914755	OAR20_40622073.1 36784515	13.28	0.02
24	1	s72739.1 101606	s12995.1 7650690	10.36	0.02

**Figure 3:**
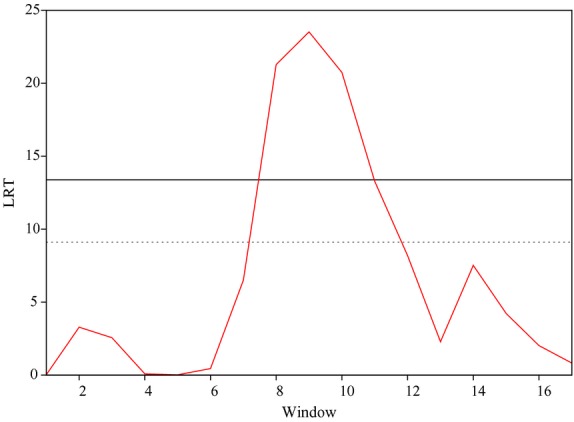
Plot of the likelihood ratio test (LRT) across chromosome 20, considering the n-1 chromosome genomic relationship matrix for *Strongyles* average animal effect. Genome-wide p < 0.05 (solid line) and suggestive (dashed line) thresholds are also shown.

Figure 3 shows the LRT profile for OAR20. These results indicate that significant effects are observed for a small number of windows on each side of the peak; however, the LRT values quickly decay to negligible as you move further from the peak. It should be noted that for nearly all windows in every analysis, apart from those where a peak was observed, the region-specific heritabilities were negligible (i.e. the 75% of windows has a heritability <0.001).

**Figure 4:**
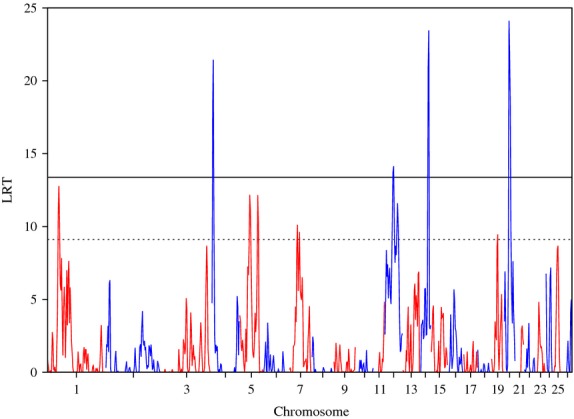
Plot of the likelihood ratio test (LRT) across the genome, considering the n-1 chromosome genomic relationship matrix for data combining *Nematodirus* for Scottish Blackface population and *Strongyles* for Sarda × Lacaune and Martinik Black-Belly × Romane populations. Genome-wide p < 0.05 (solid line) and suggestive (dashed line) thresholds are also shown.

The analysis where *Nematodirus* and *Strongyles* data were combined also identified the regions on OAR 4 and 20 found in the previous analyses, and detected two regions on OAR 12 and two on OAR 14 reaching the genome-wide significance level (Figure 4 and Table 3).

**Table 3:** Regional heritability (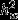) considering the n-1 chromosome genomic relationship matrix for data combining *Nematodirus* for Scottish Blackface population and *Strongyles* for Sarda × Lacaune and Martinik Black-Belly × Romane, for windows significant both at the genomic level (p < 0.05) and at the suggestive level

OAR	Window	SNP and position (in bp)		LRT	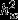
		Start	End		
4	2	OAR4_3821431.1 3731353	OAR4_11023787.1 10238576	18.38	0.02
4	3	OAR4_7316477.1 6895493	OAR4_13768293.1 12959670	21.44	0.02
12	13	OAR12_39896412.1 35380248	OAR12_46545889.1 41455928	13.72	0.01
12	14	OAR12_43172807.1 38463864	s74913.1 44383517	14.14	0.01
14	13	s18934.1 42569443	OAR14_52929077.1 49757018	21.08	0.02
14	14	OAR14_48339683.1 45853966	OAR14_57689791.1 54436843	23.46	0.03
20	8	s75212.1 21544707	OAR20_29673753.1 27071832	24.12	0.02
20	9	OAR20_25310480.1 24023600	s02808.1 30879395	22.06	0.02
20	10	OAR20_29777242_X.1 27178392	OAR20_37979703.1 34257323	18.34	0.02
1	17	OAR1_51923225.1 49582895	OAR1_59033021.1 55894733	10.82	0.01
1	18	OAR1_55194024.1 52549191	s57947.1 59090067	12.78	0.01
1	19	OAR1_59062804.1 55900976	s57980.1 62262023	10.34	0.01
4	4	OAR4_11050095.1 10264337	OAR4_16557691.1 15678300	9.76	0.01
5	16	OAR5_52221425.1 48431395	OAR5_58968886.1 54639928	12.18	0.01
5	17	OAR5_55493305.1 51654379	s06427.1 59032331	11.02	0.01
5	28	OAR5_92603004.1 84918234	OAR5_98355816.1 90702581	12.16	0.02
5	29	OAR5_95145531.1 87587746	OAR5_100913555.1 93303039	10.10	0.01
7	13	OAR7_38845979.1 34448441	OAR7_43867453.1 39423974	10.12	0.01
7	16	OAR7_47757823.1 42931982	OAR7_53605138.1 48351746	9.62	0.01
12	12	OAR12_37241680.1 32919247	OAR12_43103127.1 38396797	10.68	0.01
12	15	s04690.1 41497406	s21761.1 47009181	9.70	0.01
12	19	OAR12_60135274.1 54027615	s50610.1 59498519	9.78	0.01
12	20	OAR12_63132677.1 56732372	OAR12_68810185.1 62292818	11.60	0.01
12	21	s67454.1 59515159	OAR12_71559303.1 65064742	10.00	0.01
19	10	OAR19_31723606.1 30252049	OAR19_38703754.1 37090315	9.46	0.01
20	11	OAR20_34312897.1 30914755	OAR20_40622073.1 36784515	9.60	0.02

## Discussion

Analysis of our three genetically distant sheep populations, with contrasting experimental protocols for phenotype collection, identified regions affecting FEC not previously revealed in the individual studies, demonstrating the power of meta-analysis over single analyses. However, in this study, we also showed that results can be affected by the methodology used (RHM with either whole or n-1 **G** matrix) and the trait definition (only *Stongyles* or combination of *Strongyles* and *Nematodirus*). For example, when analysing our *Stongyles* data with RHM, using the whole **G** matrix, the only significant regions were identified on OAR4 and 20. These same regions were confirmed in the other two analyses with RHM (i.e. *Strongyles* data with n-1 **G** matrix and data combining *Nematodirus* and *Strongyles* with n-1 **G** matrix), with these analyses also identifying other regions significant at both genome-wide and suggestive level.

Our results highlight the utility of using the RHM approach, which allowed us to combine the three datasets and simultaneously account for the population substructure. We achieved this by assuming no genomic covariance between the populations, and employing n-1 **G** matrices. Furthermore, this approach can detect different types of QTL architecture from LA or LD methods, as it does not necessarily require a large difference between contrasting loci/haplotypes in the same population. This approach allows identification of regions that, as a whole, contain variation which is associated with the phenotype. Therefore, a combination of small effects in the same region can also give a significant result. This may include mutations in different linkage phases with SNPs in different populations, or even different mutations in the same or different genes in the same region, affecting the outcome phenotype in the different populations. Therefore, our results may be interpreted as showing that there are potentially a number of common pathways that are underlying parasite resistance to different species.

One of the issues encountered in this analysis was that very few sires contributed to some of the subpopulations, leading to long stretches of LD in the progeny. This population structure phenomenon means that the **G** matrix simply absorbs most of the true region-specific effect, as the long stretches of chromosome which surround the window of interest are in strong LD with the window, and hence, much of the QTL effect gets partitioned into the **G** matrix rather than the window. However, this problem was partially overcome by using the n-1 **G** matrix. As demonstrated in our results using this approach, we were able to identify more significant regions, some of which were previously reported in the individual studies. Notable is the OAR12 QTL which was identified from a breed cross and was significant using LDLA techniques (Sallé *et al.* 2012), but was only found with the n-1 **G** matrix approach here (at the suggestive level with the *Strongyles* data and at the genome-wide level with the data combining *Nematodirus* and *Strongyles*). In this case, only four F0 sires from the ‘resistant’ breed carrying the QTL were used when creating the backcross population, leading to a small number of long sire-derived haplotypes with the QTL. Deleting OAR12, which carries the QTL, when correcting for polygenic effects, led to the QTL re-appearing, as it was not swallowed by the polygenic component.

It is interesting to highlight that all windows identified by RHM as significant, either at the genome-wide or the suggestive level, accounted for low heritability (i.e. 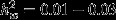), suggesting that nematode resistance is a complex trait for which many variants contribute. This is in agreement with Kemper *et al.* (2011) and Lee *et al.* (2011), who reported that there is no single mechanism of nematode resistance in sheep. Lee *et al.* (2011), however, also highlighted that the complex heterogeneity of results can be due to differences in environmental conditions, nutritional status of the animals, geographical locations and the large variety of gastrointestinal nematode parasites. However, this diversity is found in our combined dataset, and despite this diversity, significant regions were still found.

Nagamine *et al.* (2012) have reported that if a single SNP is in complete association with the causative variant (i.e. *r*^2^ = 1), then the standard single SNP-based association analysis should be the most powerful analytical approach. However, when association with any single SNP is incomplete and there may be several causative alleles, some of which are potentially rare, RHM may be advantageous. This therefore suggests that when a region contains one or more causative variants that cannot be explained by association with a single SNP, this can still be detected by the composite measure that the RHM estimate represents. The genomic architecture of the QTL will also influence the optimal window size, with smaller windows tending to be more appropriate for single causative variants, and larger windows for more complex architecture. Although a single window size was used in this study, this issue was explored by Riggio *et al.* (2013) in which the current window size of 100 SNPs was generally found to be robust. However, in the face of unknown genetic architecture, it is difficult to make strong conclusions on optimal window sizes.

All our RHM analyses results confirmed a region on OAR20 encompassing the major histocompatibility complex (*MHC*). Previously, only within SAR population, analyses identified a significant region on OAR20, close to the *MHC* (Casu *et al.* 2013), and even this result was not as significant as found in the current study. The association of *MHC* with differential response to infection has been attributed to the involvement of *MHC* gene products, leading to the induction and regulation of the immune response. However, although several studies have implicated the variation within *MHC* as a determinant of host resistance and/or sensitivity to gastrointestinal parasitism in sheep (Schwaiger *et al.* 1995) and other species (Behnke *et al.* 2003), the published results are still somewhat inconsistent and dependent on the method of analysis. For example, Beh *et al.* (2002) found no significant linkage of the *MHC* and resistance to *Trichostrongylus colubriformis*. However, it has to be highlighted that this region is characterized by extreme complexity. In humans, it has been reported that identifying casual variants in the *MHC* is uniquely challenging because the association may be with an extended haplotype that spans hundreds of genes (Vandiedonck *et al.* 2011). These authors also found that alternative splicing is extremely common for *MHC* genes, with some of the alternative splicing being haplotype specific, suggesting that alternative splicing is an additional layer of diversity in the *MHC* region. Standard SNP associations, therefore, have little chance of picking up consistent effects. On the other hand, RHM across populations is potentially more successful, as it just combines general evidence of an effect within a region.

The OAR 14 region identified as significant is the region identified by Riggio *et al.* (2013) when analysing *Nematodirus* FEC in the SBF population. However, this region did not reach significant threshold in either of the other two populations considered, nor when only the *Strongyles* data for the three populations were analysed, suggesting therefore that this region is related to the *Nematodirus* infection. This region has now been verified in three separate studies (Davies *et al.* 2006; Matika *et al.* 2011; Riggio *et al.* 2013), across several breeds (SBF, Texel and Suffolk), and represents one of the most convincing regions associated with resistance to nematode infections. In addition, a selection sweep with a medium signal around 40–55 Mbp has been identified in this region in New Zealand Texel and New Zealand Romney sheep (Fariello *et al.* 2013). These authors highlight that these two breeds are not historically closely related, suggesting therefore that the selection signature could be due to a common recent selection pressure on the two breeds and that resistance to nematodes could be one possible underlying trait associated with it. Two candidate genes have been identified in this region: *IRF3* (interferon regulatory factor) and *TGF-B1*(transforming growth factor beta-1). The *IRF3* had been already mapped to OAR14 by Jann *et al.* (2009), in a comparative genomics study of toll-like receptor signalling in five species (human, mouse, pig, cattle and sheep). These authors located *IRF3* to a region affecting health traits in the five species considered, with host QTL controlling a wide range of pathogens, making it a candidate gene for our OAR14 QTL. *TGF-B1* is important in the regulation of immune responses and plays, together with IL-10, a central role in minimizing pathology and enhancing tissue repair during helminth infections (Belkaid *et al.* 2006). Significantly positive correlation of TGF-B1 levels with adult worm count and FEC have been reported in the abomasal mucosa by Gossner *et al.* (2012) in progeny of the SBF sheep in this study.

At present, there appear to be no obvious candidate genes within the other regions identified as significant, using either the UCSC genome browser (http://genome.ucsc.edu/) or the CSIRO sheep genome browser (http://www.livestockgenomics.csiro.au/cgi-bin/gbrowse/oarv3.1/).

In summary, this study has been successful in detecting QTL for nematode resistance, combining data from three genetically distant sheep populations, identifying associations not previously revealed in the individual studies and therefore confirming the power of meta-analysis over single-experiment analyses. Moreover, by combining data from *Nematodirus* and *Strongyles* species, our results showed that potentially, there are a number of common pathways that are underlying resistance to widely different parasite species. The most significant result was in the MHC region, this being a region in which it is difficult to detect significant results using standard association analyses. This study also showed that in such situations where different populations with different genetic structures are used, and where genetic architecture of the trait may be complex, the RHM approach may be advantageous.

## Supporting Information

Data S1 Quality control (QC) measures, specific for each population.

Figure S1 Plot of principal components (PC) 1 and 2 for the three populations considered. Coloured by population, with red being the Scottish Blackface, blue the Martinik Black-Belly × Romane backcross, and green the Sarda × Lacaune backcross.
