# Supplementary material for: A joint analysis to identify loci underlying variation in nematode resistance in three European sheep populations
Source: J Anim Breed Genet. 2014 Jan 8;131(6):426–36. doi: 10.1111/jbg.12071 (PMC4258091; doi:10.1111/jbg.12071)
Supplement: Data S1 — Quality control (QC) measures, specific for each population. [file jbg0131-0426-SD1.doc]

**Supporting File 2:** Quality control (QC) measures, specific for each population.

For the Scottish Blackface, the QC removed the SNPs with a minor-allele frequency < 0.02 or a call rate < 95%. Deviation from Hardy–Weinberg equilibrium was not considered as a method for excluding SNPs. Furthermore, markers on the sex chromosome and markers unmapped were removed from the analysis (Riggio et al. 2013, Heredity 110: 420-429).

For the Martinik Black-Belly x Romane backcross, individuals with a call rate below 98% were discarded, and a 99.9% technical reliability was established by duplicated genotyping in 50 animals. In addition, Mendelian inconsistencies (i.e., no allele shared in common between a progeny and its sire for a given SNP) were checked. Intrinsic SNP quality criteria were also considered. Useless SNP that had been eliminated in the frame of the sheep HAPMAP project (i.e., SNP that Illumina annotated as abnormal; SNP with minor allele frequencies equal to 0; SNP that displayed discordant genotypes between experiments; or SNP showing Mendelian inconsistencies within the International Mapping Flock) were discarded (J. W. Kijas, personal communication). SNP with a call rate < 97% were removed. A minor allele frequency < 1% was applied. A test for Hardy-Weinberg disequilibrium was also considered to eliminate SNP with abnormal behavior (P < 10–6) in purebred populations, and these SNPs were also eliminated from the backcross genotype data. Furthermore, SNPs for which more heterozygotes than expected (i.e., 50% for a heterozygous sire) were counted, or SNPs with too great a recombination rate were not included in the QTL analysis. Sex chromosomes were not considered for analysis (Salle et al. 2012, JAS 90:4690-4705).

For the Sarda x Lacaune backcross, individuals with a proportion of genotypes missing > 10% were excluded. All SNPs which could not be mapped or were on the sex chromosome were excluded from the data set. The SNPs with call rate < 95% and minor allele frequency < 1% were also excluded. Due to the family structure of the population, deviation from Hardy–Weinberg equilibrium was not considered.
