# Supplementary material for: A joint analysis to identify loci underlying variation in nematode resistance in three European sheep populations
Source: J Anim Breed Genet. 2014 Jan 8;131(6):426–36. doi: 10.1111/jbg.12071 (PMC4258091; doi:10.1111/jbg.12071)
Supplement: Figure S1 — Plot of principal components (PC) 1 and 2 for the three populations considered. Coloured by population, with red being the Scottish Blackface, blue the Martinik Black-Belly × Romane backcross, and green the Sarda × Lacaune backcross. [file jbg0131-0426-SD2.doc]

**Supporting File 1:** Plot of principal components (PC) 1 and 2 for the three populations considered. Coloured by population, with red being the Scottish Blackface, blue the Martinik Black-Belly x Romane backcross, and green the Sarda x Lacaune backcross.
